# Using giant African pouched rats to detect tuberculosis in human sputum samples: 2010 findings

**DOI:** 10.4314/pamj.v9i1.71204

**Published:** 2011-07-18

**Authors:** Amanda M Mahoney, Bart J Weetjens, Christophe Cox, Negussie Beyene, Georgies Mgode, Maureen Jubitana, Dian Kuipers, Rudovic Kazwala, Godfrey S Mfinanga, Amy Durgin, Alan Poling

**Affiliations:** 1 Anti-Persoonsmijnen Ontmijnende Product Ontwikkeling (APOPO), Morogoro, Tanzania; 2 Sokoine University of Agriculture, Morogoro, Tanzania; 3 National Institute of Medical Research, Dar es Salaam, Tanzania

**Keywords:** Giant pouched rats, Tuberculosis detection, disease, operant conditioning, Africa

## Abstract

Giant African pouched rats previously have detected tuberculosis (TB) in human sputum samples in which the presence of TB was not initially detected by smear microscopy. Operant conditioning principles were used to train these rats to indicate TB-positive samples. In 2010, rats trained in this way evaluated 26,665 sputum samples from 12,329 patients. Microscopy performed at DOTS centers found 1,671 (13.6%) of these patients to be TB-positive. Detection rats identified 716 additional TB-positive patients, a 42.8% increase in new-case detection. These previously unreported data, which extend to over 20,000 the number of patients evaluated by pouched rats in simulated second-line screening, suggest that the rats can be highly valuable in that capacity.

## Introduction

According to the World Health Organization, about one-third of the world's population is currently infected with the *Mycobacterium tuberculosis*, which causes tuberculosis (TB), and an estimated 1.7 million people died from TB in 2009. The prevalence of TB is very high in East Africa compared to the rest of the world and TB causes many deaths and much suffering in East Africa [1].

The most common method for identifying TB in East Africa is sputum smear microscopy. This technique, though relatively inexpensive, is time-consuming and fails to detect a substantial number of TB-positive cases when large numbers of samples are analyzed [2, 3]. Scent detection of TB using African pouched rats (*Cricetomys gambianus*) has been evaluated by researchers in Tanzania. An initial proof-of-principle study suggested that the rats can detect TB-positive sputum samples quickly and more accurately than microscopy [4] and in 2009 researchers reported that scent detection rats increased new TB-positive patient findings by 31% over microscopy alone [5]. A recent study, involving 10,523 patients from Direct Observation of Treatment Short-Course (DOTS) centers in Dar es Salaam, Tanzania showed that use of the rats in simulated second-line screening during 2009 increased case detections by 44%, from 1,403 to 2,023 [6]. Each sputum sample was evaluated by 10 rats and considered positive if at least two of them identified it as such. The present study presents original data gathered during 2010 with an additional 12,329 patients. We report herein the results of that screening. The importance of the present study is that it expands to over 20,000 the number of patients evaluated by the rats in simulated second-line screening and evaluates whether results comparable to those previously reported [6] can be replicated under conditions somewhat different from those of the original investigation. Specifically, some of the rats used in the original investigation were used in the present study, but approximately 30% of the total animals were new and they were used in different configurations in the present study. That is, in the first study a sample was considered to be TB-positive if 2 of 10 rats in a group identified as TB positive. Different groups were used in the present study and in some cases the groups comprised 8 or 9, rather than 10, rats. Thus, the present study is a systematic replication and extension of the initial one.

## Methods

In 2010, the rats analyzed sputum samples obtained from DOTS centers and their evaluations were compared to evaluations by microscopy at the DOTS centers. Sputum samples were gathered from patients at the DOTS centers and then used to prepare smear slides that were stained using the standard Ziehl Neelson (ZN) method. Remaining sputum was frozen and sent to Anti-Persoonsmijnen Ontmijnende Product Ontwikkeling (APOPO) to be evaluated by the rats. APOPO obtained ethical clearance to conduct this research from the Tanzanian National Institute for Medical Research. The details of training and evaluation are described elsewhere [4, 7]. In brief, the sputum samples were sorted by APOPO technicians and heat-inactivated for 30 min at 90°C to destroy infectious microorganisms. Ten pots, each containing a single sputum sample, were simultaneously presented to the rats by placing those pots below holes equally spaced along the centerline of floor of a testing cage 205 cm long, 55 cm wide, and 55 cm high [4]. During test sessions, a rat walked along the floor of the cage, sniffing each sample. An indication response was scored when the rat paused with its nose over the sample for 5 seconds. Immediately after an indication on a known positive sample (i.e., a sample deemed TB-positive at a DOTS center), a small amount of banana mixed with crushed commercial rat chow was delivered. Indications on all other samples had no programmed consequences.

Each patient supplied either two or three samples that were evaluated by 8, 9, or 10 rats. The number of rats varied because small experiments involving some of the rats were embedded in the overall project described here. Sputum samples that were evaluated as TB-negative by the DOTS centers but TB-positive by at least two rats were re-evaluated by a technician at APOPO using ZN or FM microscopy. The “sister samples,” or the second sample from a single patient, were reevaluated as well. Patient samples that were found TB-negative by a DOTS center but APOPO ZN- or FM-positive were deemed new case detections. A list of new cases was generated every Friday and reported to the appropriate DOTS Centers for follow-up.

## Results

**Figure 1:**
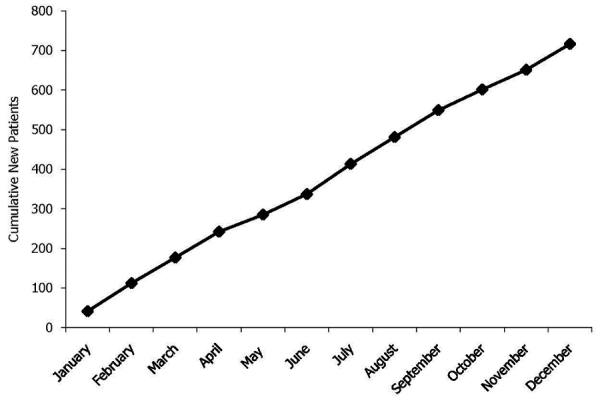
The cumulative number of new case detections per month at APOPO (Anti-Persoonsmijnen Ontmijnende Product Ontwikkeling)

Figure. 1 shows a cumulative graph of the new case findings in 2010. All samples that were evaluated at APOPO between January 1^st^ and December 31^st^, 2010 were included. Monthly new case detections ranged from 41 (January) to 76 (July), with a total of 716 new cases of TB found. The mean value was 59.4 new cases per month. In total, 26,665 sputum samples were evaluated from 12,329 patients. DOTS microscopists found 1,671 (13.5%) of the patients to be TB-positive. The rats as a group identified 1,574 of these patients as TB-positive with a positive sample defined as an indication by at least two rats. The rats also identified as TB-positive 3,844 DOTS negative patients. APOPO's microscopists confirmed the presence of *Mycobacterium tuberculosis* in 716 of these patients. Thus, the use of rats in simulated second-line screening increased the new-case detection rate by 42.8%.

Overall, there were 22,858 DOTS-negative samples. Of these, 5,421 were indicated as positive by at least two rats but were found negative by a second microscopy at APOPO. There were 3,807 DOTS-positive samples and of these, 418 were not indicated by at least two rats but were found positive by a second microscopy at APOPO. Thus, when multiple rats evaluated the same sample with a positive sample defined as an indication by at least two rats, the overall sample-wise sensitivity relative to the combined results of DOTS and APOPO microscopy was 89% and specificity was 76.3%. The overall patient-wise sensitivity was 95.6% and specificity was 73.6%.

Sample-wise sensitivity and specificity were also calculated for 10 individual rats that were operational for all of 2010 and were not included in any experiments (Table 1). Relative to DOTS-center and APOPO microscopy combined, average specificity was 85.7% (range 82.2-89%) and average sensitivity was 72% (range 69.2-76.8).

**Table 1:** Performance of individual rats

Rat	Sensitivity^α^	Specificity^α^
1	72	87
2	72	85
3	69	89
4	77	82
5	71	82
6	70	88
7	77	82
8	72	87
9	71	86
10	69	89

α Relative to the results of the DOTS center and APOPO (Anti-Persoonsmijnen Ontmijnende Product Ontwikkeling) microscopy

## Discussion

Detection rats increased new case detections by 44% in 2009 [6] and by 43% in the current study, which reported results from 2010. Very similar new case detections were found in the two studies, which report entirely independent data sets collected at different points in time and with different groups of rats, which suggests that the results initially reported [6] are reproducible. The present data extend to 22,870 the number of patients evaluated by pouched rats; this is a sizeable and clinically significant number. Although the present findings, like those presented earlier [4, 6], suggest that pouched rats can be useful in second-line TB screening, a skeptic might argue that the new-case detections were the result of simply exposing some samples to two analyses with smear microscopy. As we have demonstrated previously [6] the proportional increase in new-case detections is too high for this to be the case. Therefore, it appears that pouched rats may be useful in TB screening. These rats can evaluate samples faster than microscopists and are at least as accurate. Moreover, rats’ evaluations substantially increase the case detection rate compared to ZN microscopy alone.

As of yet, however, only limited data comparing the rats’ evaluations of sputum samples to the results of culturing, the “gold standard” of TB detection, have been reported. Those data, obtained with two rats, revealed sensitivities of 73.1% and 73.1% for the individual rats and 86.6% for the two combined [4]. Specificities for the individual rats were 93% and 93.8% and for the two combined it was 89.1%. These values are good relative to those obtained with smear microscopy as typically performed [2, 3] but further research involving more rats and more cultured samples is needed before definitive statements can be made regarding the rats’ accuracy as TB detectors. Such research currently is underway at APOPO.

Additional research also is needed to ascertain the actual status of samples that are identified as negative by DOTS and APOPO microscopists but positive by the rats. During 2010, the rats identified 4,635 DOTS-negative patients as TB-positive but smear microscopy confirmed the presence of *M. tuberculosis* in only 716 of those patients (15%). Given the relatively low sensitivity of smear microscopy [2, 3], it is highly likely that sputum from some of these patients, and perhaps from many of them, actually contained the bacillus. The Cepheid GeneXpert System (Cephid, Sunnyvale, California, USA), an automated device that detects *M. tuberculosis* through polymerase chain reaction analysis, provides a rapid and accurate method for detecting TB in sputum [8] and APOPO is beginning research in which rat-positive, microscopy-negative samples are analyzed with the GeneExpert, which is currently too costly to use for examining all samples. It is hoped that the use of pouched rats for initial detection followed by the GeneExpert for confirmation will substantially increase new case detections in second-line screening without a prohibitive increase in cost.

## Conclusion

Using operant conditioning procedures, Giant African pouched rats were trained to detect tuberculosis from human sputum samples. Despite its poor sensitivity, microscopy is often used for TB detection in developing countries. Patients missed by microscopy are thought to spread the disease to 10-15 other people and it is the primary cause of death in people with HIV [1]. Relative to smear microscopy, the rats’ sample-wise sensitivity was 89% and specificity was 76.3%. Patient-wise sensitivity was 95.6% and specificity was 73.6%. Detection rats identified 716 additional TB-positive patients, increasing new case detection by 42.8%. These previously unpublished results extend to over 20,000 the number of patients evaluated by pouched rats in simulated second-line screening and confirm prior reports that detection rats are a fast and cost-effective way to identify a substantial number of patents missed by microscopy.
